# Gastroprotective mechanism of *Paederia foetida* Linn. (Rubiaceae) – a popular edible plant used by the tribal community of North-East India

**DOI:** 10.1186/s12906-015-0831-0

**Published:** 2015-09-03

**Authors:** Silpi Chanda, Lokesh Deb, Raj Kumar Tiwari, Kuldeep Singh, Sayeed Ahmad

**Affiliations:** Department of Pharmacy, Jaypee University of Information Technology (JUIT), Wakhnaghat, Solan, Himachalpradesh, 173234 India; Pharmacology Laboratory, Medicinal Plants and Horticultural Resources Division, Institute of Bioresources and Sustainable Development (Department of Biotechnology, Government of India), Takyelpat Institutional Area, Imphal, 795001 Manipur India; Department of Pharmacognosy and Phytochemistry, Jamia Hamdard, New Delhi, 110062 India

**Keywords:** *Paederia foetida*, Gastroprotective, Antioxidant, North-East India

## Abstract

**Background:**

The objective of the present study is to evaluate the gastroprotective activity of *Paederia foetida* L. leaf for gastric ulcer.

**Methods:**

The methanol extract of *P. foetida* L. leaves at two different dose levels was investigated for gastroprotective potential by using Indomethacin-pylorus ligation, alcohol induced and water immersion stress induced model in rats. *In vitro* DPPH* radical scavenging activity and western blot analysis of stomach tissue from pylorus ligatures rats were also carried out. HPTLC analysis was done to understand the phyto-pharmacological relationship.

**Results:**

Methanol extract at a dose level of 100 mg/kg and 200 mg/kg body weight showed 72 and 78 % ulcer protection when compared to negative control whereas reference drug shown 82 % protection in Indomethacin-pylorus ligation model. Further, methanol extract also showed protective effect against 70 % v/v ethanol and stress induced gastric ulcer model. About 84 % protection as compared to cimetidine (85 %) was seen in western blot analysis of stomach tissue from pylorus ligatures rats. HPTLC analysis of methanol extract of *P. foetida* L. confirmed the presence of β-sitosterol. In DPPH* radical scavenging activity, the IC_50_ value was observed to be 43.52 μg/ml.

**Conclusions:**

These observations established the traditional claim and thus *Paederia foetida* could be a potent gastroprotective agent for use in future. The gastroprotective activity might be mediated by the Nrf2 mediated antioxidant and anti secretory effects.

## Background

*Paederia foetida* Linn. belonging to the family Rubiaceae (Sanskrit, Prasarini) is an extensive foetid smelling climbing plant. The aerial parts of the plant contain iridoid glycosides *viz.* asperuloside, scandoside and paederoside [1]. Different tribal communities of North East to Southern part of India use the plant as a vegetable and also use to treat different stomach disorders like diarrhoea and dysentery [2–6], stomach swelling, to clean stomach, gastritis, in loose motion, indigestion, abdominal pain etc. [3, 7–18]. The leaf is also used as an anti ulcer agent [19]. Various scientific study has been carried out to investigate the use of Paederia in the treatment of rheumatic affections [20–22], anti-inflammatory, hepatoprotective, [23–25], tripsyChemolithotripsy [26], promotes sexual vigour, increase the quantity of semen and body strength and to produce a youthful glow [27].

Peptic ulcer disease (PUD) is the most common gastrointestinal disorder in clinical practice. PUD results from an imbalance between defensive and corrosive factors, i.e. corrosive power of gastric hydrochloric acid and digestive power of pepsin as well as bile versus defensive factors, i.e. the ability of gastric and duodenal mucosa to resist these corrosive/digestive powers. Several side effects *viz.* hip fracture, acute interstitial nephritis, acute renal failure, osteoporosis, hepatitis, visual disturbance of currently available medicine for PUD has been reported [28–30]. Therefore, there is an urgent need to look for a better alternative treatment of PUD. *Helicobacter pylori* infection [31, 32] and oxidative stress [33] are one of the major cause for the development of the PUD. Our previous studies on biological activity of Paederia against *H. pylori* and oxidative stress showed promising results [34, 35]. In the light of these outcomes we further investigated the anti ulcer activity of the plant on animal models to evaluate the use of Paederia in the treatment of peptic ulcer. The present article describes our efforts and outcome.

## Methods

### Ethnobotanical survey and Plant materials collection

To know the importance, utility and therapeutic efficacy of the wild edible plants of Northeast, India, a survey [8] was conducted by the author S. Chanda. Agartala, capital of Tripura was chosen as a study area for the ethnobotanical survey. The survey was conducted randomly among the tribal families residing in and around Agartala. This ethno-pharmacological survey was carried out as per rules of Indian Biodiversity Act and Manipur Biological Diversity Rules, 2008 item No. 15(1) and 15(2), where stated that “citizen of India or corporate, association or organization registered in India under section 7 of the act are exempted from seeking prior/previous approval for the purposes of research, bio-survey and bio-utilization”. The first main criteria to chose any consultants for the current study was the deep knowledge of the consultants in the use of plants and secondly the ability to speak and understand the different tribal and non tribal languages of Tripura. A general conversation also has been done with the several tribal medicine men (traditional practitioner) of that particular area. Here medicine man (locally known as Kaviraj/Baidya) is a healer who acquired deep knowledge on the use of plants as medicine from his or her forefather and use the knowledge to treat people when they are ill. For the current study the oral consent was obtained from the traditional practitioners. Total sample was considered by approaching direct interviews with individual tribal families and medicine men. A structured unbiased questionnaire format was prepared to facilitate the outcome of list of the plants used in the treatment of stomach disorders particularly ulcer. Medicine men were questioned about their diagnostic procedure, symptoms and the method of medication to treat stomach or duodenal ulcer. The most common symptoms described by the medicine men, which were further characterized as either ulceration in stomach or risk factors to developed ulceration are acidity, feeling of a full stomach, excessive abdominal pain which relieves after food consumption, loss of appetite with abdominal pain, belching with a sour taste etc. We have promised to respective traditional practitioners that the obtained traditional knowledge and sample will used only for our research purpose and will not disclosed their typical formulation or excipients to any one. After thorough analysis of the filled questionnaire format we identified that the plant *Paederia foetida* L. is one of the most promising plant in the treatment of ulceration use by the tribal families. This plant is also very frequently advised by the medicine men to treat such disease as it is edible too. Fresh leaves of *Paederia foetida* Linn. (Rubiaceae) were collected from the local tribal market of Agartala, Tripura and authenticated by the National Institute of Science Communication and Information Resources, New Delhi (Authentication Ref No. NISCAIR/RHMD/Consult/2010-11/1442/40).

### Preparation of extract

The leaf material was collected from Agartala, Tripura and extraneous matter were removed. The leaf material was dried in shade for 10 days and ground to get course powder (about 1.5Kg). The ground material was extracted with methanol (4.5 L) in a Soxhlet apparatus for 24 h. The temperature was maintained not exceeding 70 °C throughout the extraction procedure. The methanol extract was collected, filtered and evaporated to dryness on a rotary evaporator. The completely dried methanol extract was rewashed with distilled water, redried and stored in refrigerator until use. The yield of methanol free extract was 36.21 % w/w.

### Animals

Healthy albino rats (Wistar strain) weighing 150 to 180 g were procured from Regional Institute of Medical Sciences (RIMS), Imphal. Ethical clearance for handling and experimentation on animals was obtained from the Institutional Animal Ethical Committee (IAEC), IBSD, Imphal with approval No.-IBSD/IAEC/Ext. Inst./PC/4 (1) prior to the beginning the experiment works. All studies were conducted in an accordance with the Animal Ethical Committee. The rats were acclimatized for one week under laboratory conditions to adopt in a new environment, where experiment was performed. They were housed in polypropylene cages and maintained at 25 °C ± 2 °C temperature and 12 h dark/light cycle. They were fed with soya bean *bori*, Gram or standard pellet food once daily and water ad libitum throughout the experiments. The beding materials in the cages were renewed daily to ensure hygienic condition and maximum comfort for animals.

### Determination of acute toxicity (ALD_50_)

The acute toxicity for methanol extract of *P. foetida* was determined in albino mice, maintained under standard conditions. The animals were fasted overnight prior to the experiment. Acute oral toxicity – acute toxic class method (OCED Guideline no. 423, Annexure – 2d) adopted by CPCSEA, Government of India was followed for toxicity studies. The mortality was observed after oral administration of 2000 mg/kg b.w test samples [36]. Common side effects such as, mild diarrhoea, loss of weight and depression of treated groups of animals was recorded within the 7 days of observation [37].

### Indomethacin-pylorus ligation-induced ulcer

Albino rats were randomly divided into five groups, each consisted of 5 animals. The normal control (Group 1) and ulcer control (Group 2) groups received vehicle (distilled water; 5 ml/kg, p.o.) throughout the course of the experiment. The treatment groups received different doses of methanol extracts of *P. foetida* (100 and 200 mg/kg b.w./day; Group 3 & 4) and ranitidine (10 mg/kg, p.o./day; Group 5) for a period of four days. All the animals, other than those in the normal group, were administrated with indomethacin suspension (25 mg/kg, s.c.) once daily for four days as a ulcerogenic agent, 30 min after the administration of vehicle or test or standard drugs.

#### Surgical procedure

On the 4^th^ day 30 min after the treatment, surgical procedure was done [38]. The rats were fasted for 24 h before starting of surgical procedure. They were provided free access to water during this period. Each rat was anaesthetized with ether and the abdomen was opened through a midline incision. The pylorus located and ligated tightly with silk suture. Both the muscular layers and skin were then stitched with the help of suture and ligature separately. Four hours after pylorus-ligation, the animals were sacrificed by giving over dosage of ether. Stomach of all sacrificed rats was examined under microscope. The gastric damages in the glandular regions were located in the gastric mucosa as elongated black-red lines parallel to the long axis of stomachs.

#### Calculation of ulcer index [39]

$\mathrm{Ulcer}\kern0.5em \mathrm{index}\kern0.5em =\kern0.5em \left(\mathrm{U}\mathrm{N}+\kern0.5em \mathrm{US}\kern0.5em +\kern0.5em \mathrm{UP}\right)\kern0.5em \times \kern0.5em {10}^{-1};$Where UN = Average of number of ulcer per animal

US = Average of severity score, UP = Percentage of animals with ulcers.

The percentage of ulcer protection was calculated by using the following formula:-

$$ \%\kern0.75em  of\  ulcer\  Protection = \frac{\mathrm{Control}\ \mathrm{mean}\ \mathrm{ulcer}\ \mathrm{index} - \mathrm{test}\ \mathrm{mean}\ \mathrm{ulcer}\ \mathrm{index}}{\mathrm{Control}\ \mathrm{mean}\ \mathrm{ulcer}\ \mathrm{index}}\times 100 $$

#### Determination of gastric volumes, pH and acid outputs

The stomachs of rats were excised out under ether anesthesia exactly after 4 h of pylorus ligation and the gastric contents were collected. The stomachs were washed with luke warm sterile water. Both the washing and gastric contents were collected and centrifuged together at 4000 RPM for 10 min. The volumes of all the supernatants of gastric contents were measured and other gastric secretion studies, like pH, acid output, acidity, ulcer index/ulcer score have also been done [40, 41]. The pH of all supernatants was measured and their acidities were determined by titration to pH 7 with 0.1 N NaOH solution.

The acid outputs were calculated by following equation:

$$ \mathrm{Acid}\ \mathrm{o}\mathrm{utput}\ \left(\upmu \mathrm{E}\mathrm{q}/\mathrm{hr}\right) = \mathrm{Acid}\mathrm{ity}\ \left(\mathrm{mEq}/\mathrm{l}\right)\times \mathrm{V}\mathrm{o}\mathrm{l}.\ \mathrm{o}\mathrm{f}\ \mathrm{gastric}\ \mathrm{juice}\kern0.75em \left(\mathrm{ml}\right)/4\kern0.5em \left(\mathrm{hr}\right) $$

$$ Acidity = \frac{Titre\  value\  of\  NaOH\ X\  Actual\  normality\  of\  NaOH}{Assumed\  normality\  of\  NaOH}\times 100 $$

### Alcohol induced gastric ulcer

The animals were divided into five groups, each consisting of five rats. The ulceration was induced by administering 70 % ethanol (10 ml/kg, p.o.). All animals were fasted for 36 h before administration of alcohol. Group 1 receiving distilled water (5 ml/kg, p.o.). Group 2 receiving 70 % ethanol to induce gastric ulcer and 5 ml distilled water/kg p.o. as treatment, Group 3 & 4 received methanol extracts of *P. foetida* (100 and 200 mg/Kg, p.o.) one hour before administration of 70 % ethanol as single dose. Sucralfate was administered to fifth group as reference standard drug at the dose100mg/kg, p.o. one hour before administration of ethanol. They were kept in specially constructed cages to prevent coprophagia during and after the experiment. The animals were anaesthetized 4 h later with anesthetic ether and stomach was incised along the greater curvature and ulcer score was recorded [41]. The ulcer score for the normal stomach, red coloration, spot ulcer, hemorrhagic streak, ulcer and perforation is 0, 0.5, 1.0, 1.5, 2.0 and 3.0 respectively.

### Water immersion stress induced ulcer (WISIU)

The animals were divided into five groups, each group consisting of five rats. Group 1 (normal control) received distilled water (5 ml/kg, p.o.) where as group 2 treated as ulcer control. Group 3 and 4 received methanol extracts at the dose 100 & 200 mg/kg b.w. respectively and group 5 received lansoprazole (8 mg/kg, p.o.). After treatment, animals were allowed to swim in a glass cylinder having water (25 °C) for 3 h . Animals were killed by high dose of anesthetic ether and ulcer score was recorded [42].

### Western blot analysis of in-vivo stomach tissue from pylorus ligatures rats

Animals were divided into four groups, each containing six and were starved for 24 h before experimentation. Group1 treated as normal control (+ control) and administrated water orally. The dose was 5 ml/kg b.w. Group 2 treated as an ulcer control (− control). Group 3 and 4 received *P. foetida* methanol extract (150 mg/kg body weight, p.o.) and cimeditine (8 mg/kg, body weight p.o.) respectively. After 30 min of treatment the pylorus located and ligated tightly with silk suture as described above in the indomethacin induced ulcer model. The stomachs of rats were removed and opened along the greater curvature and then washed with ice cold phosphate buffered solutions. The number of either erosions or ulcers was determined under the magnifying glass and gastric secretion studies have been done [40, 41].

Homogenates obtained from scratched gastric mucosa was centrifuged at 4000 RPM, 4 °C for 30 min (5430R, Eppendorf). The supernatants containing 1:1000 protease inhibitor cocktail (P8340, Sigma-Aldrich) were kept into −80 ° C until used. The proteins in tissue homogenates were separated by SDS-PAGE and transferred to polyvinylidene difluoride (PVDF) membranes (Merck- Millipore), by means of trans blot turbo unit (BIO RAD). The transferred proteins were incubated with primary antibodies (Anti-Nr2f2; Ab1) produced in rabbit, washed and incubated with biotin conjugated secondary antibodies. After rewashing it was visualized by chromogenic method in SNAP-ID instrument (Merck- Millipore).

### Statistical analysis

Data were expressed as mean ± Standard Error Mean (SEM). Differences were considered significant at ^***^P<0.001, or ^**^P < 0.01 or ^*^P<0.05 when compared test group vs control (−ve) group and ^ααα^*P*<0.001, or ^αα^*P* < 0.01 or ^α^*P*<0.05 when compared positive control vs negative control. For numerical results, one-way analysis of variance (ANOVA) with Dunnett test (Positive control vs negative control and all test group vs. negative control) was performed using GraphPad InStat Version 3 (GraphPad Software).

### DPPH* radical scavenging activity

Though the antioxidant activity of the fresh leaves of Paederia also has been done by the author [34] but the same was also repeated with the dried sample (methanolic extract of dried leaf) used for the current animal study. The free radical-scavenging activity of methanol extract of Paederia was measured in terms of hydrogen donating or radical-scavenging ability using the stable radical DPPH. A solution of 0.1 mM α,α-diphenyl-β-picryl-hydrazyl (DPPH) (Himedia, Mumbai) in ethanol was prepared and 1.0 mL of this solution was added to 3.0 mL of the extract solution in water at different concentrations (10–100 μg/mL). Thirty minutes later, the absorbance was measured at 517 nm [43]. The lower absorbance of the reaction mixture indicates higher free radical-scavenging activity. Rutin (Ozone, Mumbai) was used as a standard drug. The results were expressed as IC_50_ or inhibitory concentration 50 value, i.e. concentration of samples exhibited 50 % inhibition of DPPH* radicals.

### HPTLC analysis of methanol extract of *P. foetida*

The methanol extract of *P. foetida* was evaluated by using high performance thin layer chromatography (HPTLC) system (Camag linomat 5, Switzerland) for qualitative estimation of β*-*sitosterol at 540 nm. The commercially available β*-*sitosterol (Sigma-Aldrich; India) and methanol leaf extract of *P. foetida* was run simultaneously using mobile phase (toluene: ethyl acetate: formic acid in a ratio of 8:2:0.05 v/v/v) and anisaldehyde sulphuric acid used as visualizing reagent.

## Results

### Determination of acute toxicity (ALD50)

In acute toxicity study, methanol extract of *P. foetida* does not show any mortality at the dose level of 2000 mg/kg. Therefore 2000 mg/kg dose was considered as ALD_50_ cut off the dose under GHS 5 (safe dose), as per Globally Harmonised Classification System (GHS) for Chemical Substances and Mixtures described in OECD guideline 423 (Annexure 2b & 3b). Common side effects such as, mild diarrhoea, loss of weight and depression in the treated group of animals were not observed within the 7 days of observation. In the present study, only 100 mg/kg (low) and 150 mg/kg (high) or 200 mg/kg (high) body weight doses were selected for in-vivo gastroprotective study and 10–100 μg/mL doses for *ex-vivo* and *in-vitro* studies. Because, ≥200 mg/kg body weight of extracts for in-vivo studies and ≥200 μg/ml of extract concentrations in *in-vitro* are likely to be artificial despite of yielding reproducible effects. Even worse, such high concentrations may trigger non-physiological effects resulting in artifacts [37].

### Indomethacin-pylorus ligation model

In this model, administration of methanol extract of two different doses (100 and 200 mg/kg) inhibited the increase in gastric secretion volume, acid output in a dose dependent manner whereas there is an increase in pH. The effect was highly significant (p<0.001) for both the doses. The gastric ulcer index was decreased. It showed ulcer protection, 72 and 78 % at the dose of 100 and 200 mg/kg, respectively, where ranitidine showed 82 % of ulcer protection (Table 1).

**Table 1:** Effect of methanol extract on gastric secretion volume, pH, acid-output and ulcer index against indomethacin-pylorus ligation induced ulcer in rats

Treatment	Gastric secretion (Mean ± SEM)			Ulcer index (mm) Mean ± SEM	% of ulcer protection
	Volume (ml)	pH	Acid output (Eq/100 g/4 h)		
Group 1 (5 ml distilled water/kg)	2.114±0.13	2.80±0.01	2.80±0.01	2.850±0.07	-
Group 2 Indomethacin (25 mg/kg)	9.280±0.09^ααα^	1.07±0.01^ααα^	24.47±2.28^ααα^	39.12±0.81^ααα^	-
Group3 MeOH PF (100 mg/kg)	4.296±0.11^***^	2.51±0.02^***^	10.99±0.11^***^	10.57±0.15^***^	72.98
Group 4 MeOH PF (200 mg/kg)	2.670±0.21^***^	2.71±0.71^***^	7.62±0.14^***^	8.38±0.05^***^	78.58
Group5 Ranitidine (10 mg/kg)	2.426±0.52^***^	2.89±0.03^***^	5.30±0.31^***^	6.69±0.06^***^	82.89

MeOH PF= methanol extract of *Paederia foetida*; n=5; Data = Mean±SEM; ^ααα^ *P*<0.001 (G1 Vs G2); ^***^ *P*<0.001, (Group 2 Vs Group 3, 4 & 5)

### Alcohol induced gastric ulcer model

Oral administration of ethanol induces ulcer. Pretreatment of such rats with different doses of the extracts showed highly significant protection to ulceration. 59 and 72 % ulcer protection was seen in the dose of 100 and 200 mg/kg, respectively, whereas standard drug sucralfate showed 81 % of ulcer protection (Table 2).

**Table 2:** Effect of methanol extract of *P. foetida* on alcohol-induced gastric ulcer in rats

Treatment	Ulcer score	% of ulcer protection
Group 1 (5 ml distilled water/kg, p.o.)	2.0 ± 0.12	-
Group 2 (5 ml distilled water + 70 % ethanol, 10 ml/kg, p.o.)	3.70 ± 0.49 ^α^	-
Group 3 MeOH PF (100 mg/kg)	1.40 ± 0.29^*^	59.45
Group 4 MeOH PF (200 mg/kg)	1.0 ± 0.31^*^	72.97
Group 5 Sucralfate (100 mg/kg, p.o.)	0.70 ± 0.12^*^	81.08

MeOH PF= methanolic extract of *Paederia foetida*; n=5; Data = Mean±SEM; ^α^ *P*<0.05 (Group1 Vs Group2); ^*^ *P*<0.05, (Group 2 Vs Group 3, 4 & 5)

### Water immersion stress induced ulcer (WISIU) model

The methanol extract (100 and 200 mg/kg) dose dependently showed highly significant (p<0.001) ulcer protection (62 and 67 %) to stomach mucosa of rats against WISIU where as lansoprazole showed 70 % protection (Table 3).

**Table 3:** Effect of methanol extract of *P. foetida* on water immersion stress induced ulcer in rats

Treatment	Ulcer score	% of ulcer protection
Group 1 (5 ml distilled water/kg, p.o.)	2.0 ± 0.12	-
Group 2 (ulcer control)	4.00±0.22	-
Group 3 (MeOH PF 100 mg/kg, p.o.)	1.50±0.27^***^	62.50
Group 4 (MeOH PF 200 mg/kg, p.o.)	1.30±0.12^***^	67.50
Group 5 (Lansoprozole 8 mg/kg, p.o.)	1.20±0.26^***^	70.00

MeOH PF= methanolic extract of *P. foetida*; n=5; Data = Mean±SEM; ^***^ *P*<0.001, (Group 2 Vs Group 3, 4 & 5)

### Western blot analysis of stomach tissue from pylorus ligatures rats

**Fig. 1:**
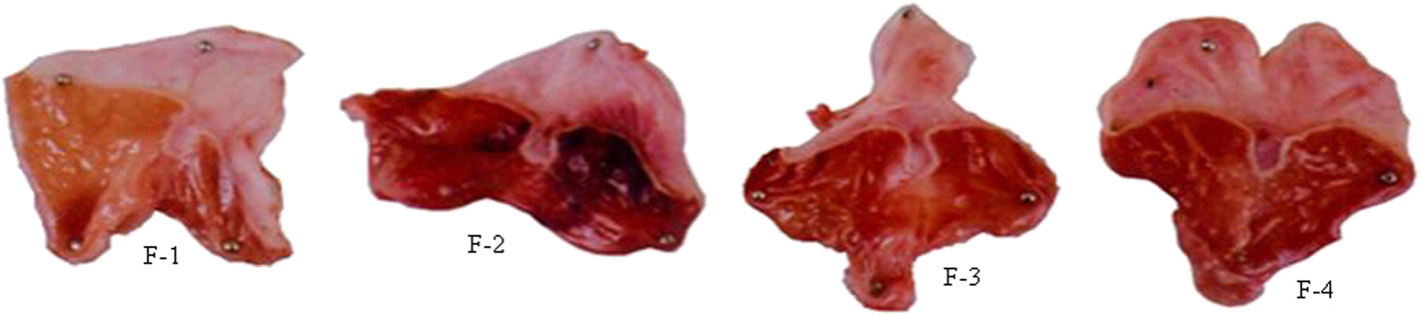
Observation of ulcers in pylorus ligatures rats; F-1: Normal control group (+ Control), F-2: Ulcer control group (− Control), F-3: *P. foetida* treated group, F-4: Cimetidine treated group

**Fig. 2:**
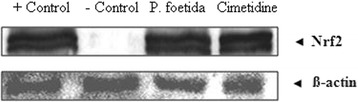
Nrf2 expression in rat stomach demonstrated by western blot analysis of pylorous ligatured rat

As reported in Table 4 and Fig. 1 (F-1 to F-4), the effects of the methanol leaf extract of *P. foetida* at a dose of 150 mg/kg, p.o. and cimetidine (8 mg/kg, p.o.) prevented the development of acute gastric ulcer. Oral administration of the extract and cimetidine decreased ulcer index to 1.11 ± 0.46 and 1.00 ± 0.23 in comparison to ulcer control 7.00 ± 0.63 (p<0.001), but acute gastric ulcer was developed significantly (p<0.001) in ulcer control when compared to the ulcer index of normal control animals 0.33 ± 0.11. Compared to the ulcer control rats, methanol extract of *P. foetida* and cimetidine pretreatment rats were shown a significant reduction in gastric volume, free acidity and total acidity (P<0.001). Figure 2 explained that in ulcer control (− control) group Nrf2 was not expressed, whereas in the normal control (+ control), methanol extract of *P. foetida* and cimetidine treated group showed expression of Nrf2.

**Table 4:** Effect of methanol extract of *P. foetida* on Pylorus-ligated rats

Treatment	Mean ulcer Index (SEM)	% Protection	Volume (ml) of gastric secretion	Free Acidity (Eq/l/100gm)	Total Acidity (Eq/l/100gm)	pH
Group 1 Normal Control (Distl. Water, 5 ml/kg, p.o.)	0.33 ± 0.11	-	1.52 ± 0.49	71.97 ± 1.04	83.23 ± 0.68	3.65 ± 0.11
Group 2 Ulcer Control (Distl. Water, 5 ml/kg, p.o.)	7.00 ± 0.63***^c^	-	7.40 ± 0.61***^c^	88.17 ± 2.14***^c^	130.17 ± 6.41***^c^	2.38 ± 0.19***^c^
Group 3 Methanol extract (150 mg/kg, p.o.)	1.11 ± 0.46***	84	2.20 ± 0.35***	50.17 ± 5.12***	60.33 ± 3.20***	5.42 ± 5.50***
Group 4 Cimetidine (8 mg/kg, p. o.)	1.00 ± 0.23***	85	1.88 ± 0.22 ***	36.00 ± 5.29***	44.00 ± 1.13***	7.42 ± 0.15 ***

Data were expressed as mean ± Standard Error Mean (SEM), where n=6 in each group. Differences were considered significant at ***P<0.001 when compared control group vs ulcer control group (c) and Test groups vs ulcer control group. For numerical results, one-way analysis of variance (ANOVA) with Tukey-Kramer Multiple Comparison post tests were performed using GraphPad InStat Version 3 (GraphPad Software)

### DPPH* radical scavenging activity

In *in-vitro* assay, the IC_50_ values of reference standard rutin and methanol extract of *P. foetida* leaf was calculated 17.97 μg/ml and 43.52 μg/ml for DPPH* radical scavenging effect respectively.

### HPTLC analysis of methanol extract of *P. foetida*

**Fig. 3:**
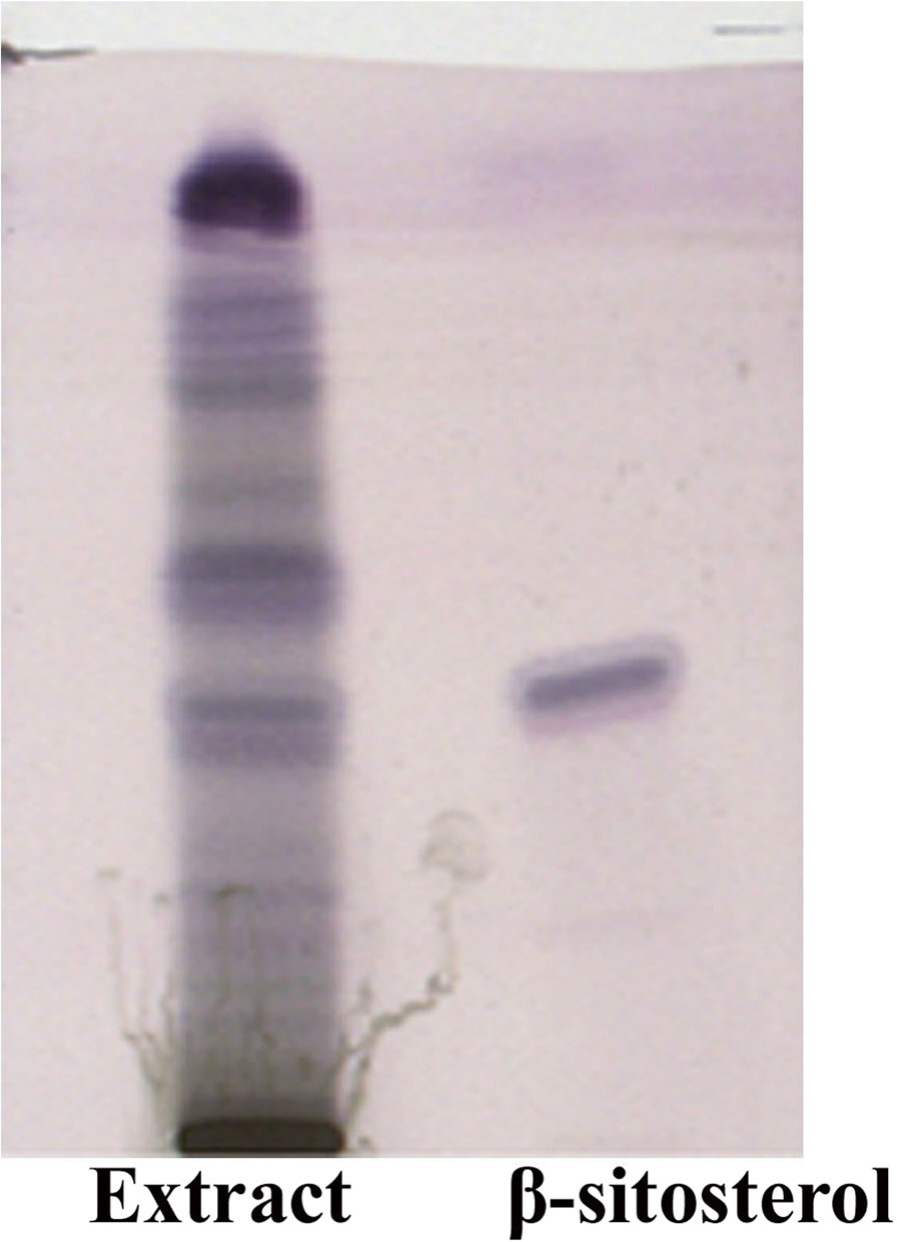
HPTLC chromatogram of *P. foetida* methanol extract and standard β-sitosterol

HPTLC showed the presence of β-sitosterol like compound in methanol extract. The R_f_ value of standard β-sitosterol compound was similar (R_f_ 0.38) with one of the compounds visualized in the chromatogram of methanol extract at 540 nm and as well as after chemical treatment (Fig. 3).

## Discussion

In our current study, we mainly focused the leaf portion of the plant on the basis of the outcome of the ethno-botanical field survey report conducted by the corresponding author [8] which revealed that leaf is only the part showing dual use as edible as well as medicine by the tribal people of Tripura. Secondly, extensive review of literature (2004 – 2012) covered by the authors’ concern to only the ethnobotanical survey report (n=24) a total of 79.17 % of articles claimed the use of leaf as ethnomedicine whereas 12.5 % (*n*=3) claimed the use of whole plant, only 8.33% (*n*=2) stated the use of root and twig and 4.17 % article claimed the use of stem, stem bark, aerial part and fruit individually. So in the present study, we focused to evaluate the Paederia leaf as gastroprotective agent on animal models. Our previous *in vitro* study [34, 35] also indicated the same. We tried to explore the mode of action which effect in physiological mechanism that caused ulcers. Though Reddy *et al.* [44] reported its anti ulcer activity of the aqueous and ethanol root extract of the plant, but there is still to go on protein based mechanism level. In the current study, western blot analysis of stomach tissue and antioxidant activity of the leaf extract explained the mechanism of gastroprotective activity. Ulcer protective activity might be mediated through Nrf2-antioxidant response element (ARE) pathway. Further, transcription factor Nrf2, a member of the Cap ‘n’ Collar family of bZIP proteins, is a central regulator in both constitutive and inducible ARE-controlled gene expression and innate immune response. Nrf2 mediated antioxidant response represents a critically important cellular defense mechanism that serves to maintain intracellular redox homeostasis and limit oxidative damage [45]. The Nrf2 expression was observed in the methanol extract of *P. foetida* and cimetidine treated groups, but not in ulcer control group. The above observations suggest the role of free radical scavenger(s) present in methanol extract of *P. foetida* induced gastro protective effect.

Uncontrolled acid secretion and ulceration of stomach mucosa due to several reasons have posed serious problems to the human health all over the globe [46]. There are several methods for the evaluation of gastric and duodenal antiulcer agents like pylorus ligation, stress ulcers, histamine, acetic acid, NSAIDs induced ulcers, reserpine induced solitary chronic gastric ulcers, serotonin-induced gastric mucosal lesions, cysteamine, dimaprit, endotoxin, dulcerozine, indomethacin plus histamine, MPTP (1-methyl-4phenyl-1,2,3,6 tetrahydro pyridine) induced duodenal ulcers etc. Here we used those models which evaluate the basic mechanism of action involved, either H_2_ antagonism or anti-secretory or proton pump hydrogen potassium ATPase inhibitors (PPIs) or cytoprotection etc.

Indomethacin inhibits the activity of cyclo-oxygenase and decreases the formation of prostaglandins (PGs) [47–50], whereas it does not inhibit the lipoxygenase pathway of arachidonic acid. The increased level of LTs in gastric mucosa has potent action on the mucosal vasculature [51]. Lipid mediators play an important role in causing inflammation and pain. Indomethacin potentiates the secretary response elicited by histamine due to the inhibition of PGE_2_ synthesis. Therefore, induction of gastric ulcers and erosions is accompanied by progressive decrease in the level of PGE_2_ [52, 53]. In indomethacin-pylorus induced ulcer model methanol extract significantly decreased the gastric secretion volume, acid out-put and ulcer index while gastric pH was increased significantly in treated rats. This model indicates its anti secretary mechanism and revealed that the methanol extract of Paederia must contain active gastroprotective constituents as they effectively antagonized the gastric ulcerogenic effects.

Alcohol causes erosion and necrosis of superficial epithelial cells on gastric mucosa. Gastric damage in rats may be due to the production of leukotrienes and also involvement of 5-lipooxygenase. Prostaglandin also plays a role in alcohol induced ulcer [54, 55]. When compared with a positive control, decrease in mucosal resistance is considered to be the most important etiological reason in alcohol induced gastric ulcers. Even, there are reports that alcohol increases the secretion of protein into the gastric juice. In addition, alcohol develops ulceration by producing toxic oxygen derived free radicals [56]. Therefore, a cytoprotective agent that is capable of preventing the necrosis of mucus cell will be effective in this model, hence as a reference standard drug we chose sucralfate (a protective) not any anti secretory nor PPIs. On the other hand, in indomethacin model we chose ranitidine as a reference drug. In this model ulcer developed due to the more secretion of acid, so an antisecretory agent will be more effective rather than any protective agents.

The HPTLC analysis of the methanol leaf extract of Paederia reveals the presence of β-sitosterol in the extract that may play role in the ulcer protective mechanism. β-sitosterol was already reported for its antigastroulcerative activity [57] and anti-inflammatory properties thus it could be involved in anti ulcer property [58]. The presence of this bioactive also concrete the hypothesis of scavenging oxygen derived free radicals, generated due to alcohol, as literature supports its free radical scavenging activity also [59, 60].

Gastroprotective effect of *P. foetida* is further confirmed by WISIU model, in this case the lesion form due to both emotional and physiological stress. Lansoprazole is used to evaluate its proton pump inhibitor mechanism. The model showed almost similar ulcer protection (67 %) as standard drug lansoprazole (70 %). Despite the introduction of highly effective H_2_ blockers and proton pump inhibitors, an effective anti-ulcer drug yet to discover, which will be not only act on one basic mechanism but also should have multiple mechanism of action to heal the peptic ulcer as well as effectively prevents their recurrence. The above observation suggested multiple mechanism and ulcer protective action of methanol leaf extract of *P. foetida.* This may be due to presence of multi-functional mixture of component in the extract. The study also justified the scientific use of *P. foetida* in traditional practice for the treatment of the stomach ache and we can outlook it for the development of new potential gastroprotective agents.

## Conclusion

The present *in vivo* activity established the scientific basis of using of *P. foetida* in the treatment of peptic ulcer. The current investigation also concretes our previous *in vitro* outcomes. The ulcer protective activity of *P. foetida* might be mediated by the cholinergic action of Nrf2 mediated antioxidant and anti secretary effects*.* The antigastroulcerative activity, anti-inflammatory properties of β-sitosterol present in the methanol extract might also contribute a vital role in ulcer protective effects of *P. foetida*. However, efforts are on to investigate the role of main active principles present in the extract which are responsible for the ulcer protective activity.
